# New insights into old methods for identifying causal rare variants

**DOI:** 10.1186/1753-6561-5-S9-S50

**Published:** 2011-11-29

**Authors:** Haitian Wang, Chien-Hsun Huang, Shaw-Hwa Lo, Tian Zheng, Inchi Hu

**Affiliations:** 1 Department of Information Systems, Business Statistics, and Operations Management, Hong Kong University of Science and Technology, Clear Water Bay, Kowloon, Hong Kong; 2 Department of Statistics, Columbia University, 1255 Amsterdam Avenue, New York, NY 10027, USA

## Abstract

The advance of high-throughput next-generation sequencing technology makes possible the analysis of rare variants. However, the investigation of rare variants in unrelated-individuals data sets faces the challenge of low power, and most methods circumvent the difficulty by using various collapsing procedures based on genes, pathways, or gene clusters. We suggest a new way to identify causal rare variants using the *F*-statistic and sliced inverse regression. The procedure is tested on the data set provided by the Genetic Analysis Workshop 17 (GAW17). After preliminary data reduction, we ranked markers according to their *F*-statistic values. Top-ranked markers were then subjected to sliced inverse regression, and those with higher absolute coefficients in the most significant sliced inverse regression direction were selected. The procedure yields good false discovery rates for the GAW17 data and thus is a promising method for future study on rare variants.

## Background

There is a growing interest in the role of rare variants in disease etiology—rare in the sense that the minor allele frequency (MAF) is less than 1%. Earlier genome-wide association studies identified risk loci that accounted for only 5–10% of disease heritability [1]. There is now an increasing body of evidence that suggests an association between the rare variants and complex diseases [2]. However, the small variance of rare variants makes their association with phenotypes difficult to detect. To increase the detection power of such associations, most existing methods collapse the rare variants using biological information. Some of these collapsing methods are based on genes or pathways, whereas others involve functionality, synonymous single-nucleotide polymorphisms (SNPs), or nonsynonymous SNPs [3]. Although the collapsing methods increase the allele frequency so that the risk effect is amplified, the noise in the collapsed variables may also increase. This could render the collapsing method less effective in some cases.

In this paper, we propose a three-step method that does not use collapsing. After removing the SNPs that are identical in value across all subjects in the data, we calculate the *F*-statistic for all the markers. We show that the *F*-statistic does not down-weight a rare variant despite its low allele frequency and thus is effective in capturing the effect of the rare variant. Next, we apply sliced inverse regression (SIR) to top-ranked markers selected by the *F*-statistic. When the number of selected top markers is not too large, we find that SIR performs well in identifying the SNPs used to simulate the Genetic Analysis Workshop 17 (GAW17) phenotypes from the top markers (hereafter these SNPs are referred to as the answers).

## Methods

### Data set

The data set [4] from GAW17 consists of 24,487 SNPs and 697 unrelated individuals. The genotypes are real sequencing data from the 1000 Genomes Project. In particular, rare variants (i.e., MAF < 1%) make up 74% of the total variants. Based on the same genotypes, 200 replications are simulated. Four phenotypes are available for analysis: the quantitative traits Q1, Q2, Q4, and the disease affected status. In this paper, we use Q1 to illustrate our method, which is influenced by 39 SNPs in 9 genes. Although the method is applicable to Q2, which is influenced by 72 SNPs in 13 genes, the result is not reported here. Q4 is too noisy to obtain meaningful conclusions. Our method does not require any information on the answers, although we use the information to assess the performance of the method.

### Preliminary data reduction

Because most of the SNPs are rare variants, many of them take identical values across all individuals in the data set; that is, statistically they are indistinguishable. In the preliminary data reduction step, we remove the identical-valued SNPs and keep track of them for later reference. By doing so, we reduce the 24,487 SNPs to 15,124 distinguishable markers. The benefit of this procedure is twofold. First, the dimension is reduced for subsequent statistical analysis, and, second, removing identical-valued SNPs prevents the numerical problems caused by degenerate matrices in regression and principal component types of analyses.

### Selection by *F*-statistics

The *F*-statistic, as defined in the simple linear regression model, takes the form:(1)

where:

*Y_i_*=*β_0_*+ *β_1_X_1i_*+*ε_I_*

is the simple regression model for *i*th observation *i*= 1,…,*n* using standard notations. In particular, *ε_i_* are independent normally distributed random variables with mean 0 and variance *σ*^2^. Let  be the sample mean of *Y*’s. SSR and SSE are the regression sum of squares and the error sum of squares, respectively. The estimated model is:(2)

We calculate the *F*-statistic for each of the 15,124 markers by fitting a simple linear regression model, one marker at a time. The response variable *Y* is the average value of Q1 over 10 replications. The markers are then ranked by *F* values. A number of top markers are selected for further analysis in the next step.

### Selection by sliced inverse regression

SIR [5] is a nonparametric regression method that uses local smoothing of the response variable. It retrieves high-dimension data features from low-dimensional projection. After standardizing *X*, we estimate an inverse regression of *X* on sliced *Y*. The inverse regression, that is, the computation of *E*(*X*|*Y*), converts a high-dimensional regression problem of *Y* on *X* to many simple regressions of *X* on *Y*. In estimating *E*(*X*|*Y*), the range of *Y* is divided into small intervals (sliced) to increase computational efficiency. Next, we perform a principal components analysis on *E*(*X*|*Y*), and the principal components (PCs) are returned. These are the SIR effective dimension reduction directions discussed by Li [5]. We simply call them the SIR directions. In our analysis, SIR is performed using the R package dr.

Top markers selected from the previous step are subjected to SIR, and the response variable of SIR is the same as the one used in the previous step of determining the *F*-statistic. Because the first SIR direction (DIR1) contains the most important information of the data, it is the most significant, and we use only this SIR direction in our method. Next, we perform a two-mean clustering analysis on the absolute DIR1 coefficients. The markers in the cluster with higher mean are our final candidates for causal SNPs.

## Results

Our method is first applied to data from replicates 1 to 10 (group 1). To assess the false discovery rate (FDR) of our method, we divided the 200 replications of the GAW17 data set into 20 groups with 10 replicates per group (i.e., replicates 11 to 20 are group 2, replicates 21 to 30 are group 3, etc.). We average the ratios of the number of identified answers to the number of final candidate SNPs over 20 groups to obtain the FDR.

### Performance of the *F*-statistic

For the group 1 data, there are eight answers in the top 100 markers ranked by the *F*-statistic (Table 1), and seven of them are in the top 32. The identified answers come from four different genes, and their MAFs range from 0.1% to 6.7%. We also identified rare variants with only one minor allele (C4S1877 and C4S1889), and the *β* (influence level) of the answers were all relatively high. This result shows that the answers found by the *F*-statistic have strong main risk effects.

**Table 1:** Answers in the top 100 markers identified by the *F*-statistic in the group 1 data

Collapsed sequence	Original sequence	Gene	Rank in selected markers	SNP	MAF	*β*
10648	16705	*FLT1*	1	C13S523	0.066714	0.64997
10647	16704	*FLT1*	2	C13S522	0.027977	0.6183
10640	16692	*FLT1*	11	C13S431	0.017217	0.74136
571	5386	*KDR*	17	C4S1877	0.000717	1.07706
571 (identical)	5392	*KDR*	17	C4S1889	0.000717	0.94133
10649	16706	*FLT1*	28	C13S524	0.004304	0.62223
994	1153	*ARNT*	32	C1S6533	0.011478	0.5619
3627	5390	*KDR*	66	C4S1884	0.020803	0.29558

### How many top markers should be used?

We need to select a number of top markers for further analysis. First, the *p*-value of DIR1 reported by the SIR provides us with a clue regarding an upper bound for the number of markers to be selected. In the group 1 data, with 70 markers or less, the *p*-value of DIR1 are all well below 0.1%, but with 80 markers the *p*-value rises by more than 20 times, to 2%. This suggests that the number of markers that needs to be used should be at most 70. Second, the histograms of the *F*-statistic show a clear gap at about the top 30th position for most of the 20 groups. Thus we use the top 30th marker as the cutoff position. The results of the other cutoff positions are also calculated (Table 2). We observe that there is a trade-off between the FDR and the number of answers found. The number of answers increases with the number of markers used; however, the FDR also increases. Selecting the top 30 markers gives the best balance between the two. The performance of the proposed method for each of the 20 groups is shown in Table 3. In this case, the FDR is 20.8% and the average number of answers found is 4.3.

**Table 2:** Trade-off between FDR and the number of identified answers

Number of input markers to SIR	FDR (20 groups) (%)	Average number of identified answers (20 groups)
10	12.7	2.75
30	20.8	4.3
50	37.7	4.8
70	38.5	3.9
80	47.7	5

**Table 3:** Performance of SIR in 20 groups with top 30 markers FDR = 20.8%

Group	Number of identified answers (A)	Number of candidate markers (B)	Ratio of A to B (%)	(B − A)/B (%)
1	5	5	100.0	0.0
2	4	4	100.0	0.0
3	4	6	66.7	33.3
4	4	6	66.7	33.3
5	4	6	66.7	33.3
6	4	6	66.7	33.3
7	5	6	83.3	16.7
8	5	5	100.0	0.0
9	5	6	83.3	16.7
10	4	5	80.0	20.0
11	4	4	100.0	0.0
12	4	5	80.0	20.0
13	6	8	75.0	25.0
14	3	4	75.0	25.0
15	4	6	66.7	33.3
16	3	4	75.0	25.0
17	5	6	83.3	16.7
18	6	7	85.7	14.3
19	4	5	80.0	20.0
20	3	6	50.0	50.0

### Performance of SIR

**Figure 1:**
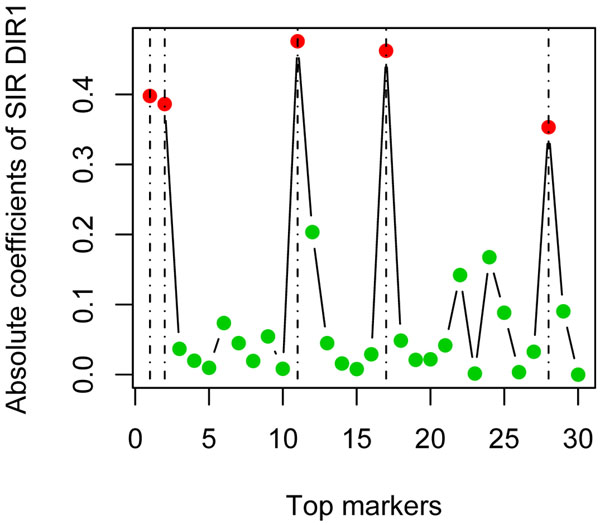
**Selection by sliced inverse regression (30 markers)**. For group 1, using 30 top markers, the absolute values of the first SIR direction coefficients are plotted in sequence. Dashed lines indicate the answer SNPs. Two-mean clustering is performed on the absolute coefficients. The red circles represent the markers in the cluster with a higher mean, which are our final selections. The green circles represent markers in the other cluster. Other groups produce similar plots. In this case, five out of five selected markers are answers.

**Figure 2:**
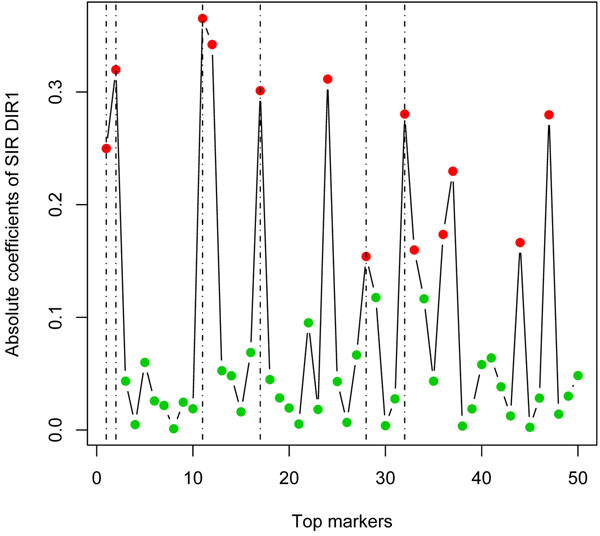
**Selection by sliced inverse regression (50 markers).** Please refer to Figure 1 for explanation. Note that, although more false positives are selected, all answers are still included among the final candidates for causal SNPs.

With the top 30 markers from the last step, for group 1, we selected five markers through SIR, and all of them are answers. Figure 1 plots the absolute coefficients of DIR1. We observe that all five answers (marked by dashed lines) reside on peak positions. A similar phenomenon is also observed in the other groups. Using more markers does bring in more false positives, but the answers still occupy the peak positions. Figure 2 shows the SIR plot using the top 50 markers. In the GAW17 meeting, many research teams reported a large number of consistent false positives with high FDRs. Our analysis shows that SIR can help to eliminate these false positives and that it has good power to identify causal SNPs.

## Discussion

### Why does the *F*-statistic work for rare variants?

We explain why the *F*-statistic works for rare variants by using a rare variant with only one minor allele (private variant). When the explanatory variables (*x*_1_, …, *x_n_*) are binary, the *F*-statistic in Eq. (1) is the same as the *F*-statistic for comparing multiple group means. We decompose the numerator into two parts: In the first part *x*_1_ is the minor allele, and in the second part *x_i_*_,_*i*=2, …, *n*, are all major alleles. The decomposition in the following equation shows the weights given to the two parts and provides an insight into the power of the *F*-statistic for a rare variant:(3)

where:(4)

The first part on the right-hand side of Eq. (3) can be written:(5)

The second part of Eq. (3) can be written:(6)

Thus the weight given to the regression sum of squares (SSR) for the minor allele is (*n* − 1) times that for the major allele. This helps to manifest the effects of rare variants on the response.

### Simultaneous versus separate treatment of common and rare variants

In the preceding analysis, we calculated the *F*-statistic for both common and rare variants simultaneously. Applying the *F*-statistic on private variants only leads to further interesting findings. After removing identical SNPs, we reduce the 9,433 private variants to 685 distinguishable markers. In the group 1 data, among the top 20 markers ranked by *F*-statistic, five answers are found. Three of them are new (CAS4935, C4S1873, and C4S1887) to the previously identified SNPs when applying the *F*-statistic to all markers. This suggests that a separate treatment of common and rare variants is a promising strategy for further investigation.

## Conclusions

We use traditional statistical methods for new applications in the context of rare variant research. A three-step method is introduced. First, we perform a simple data reduction by removing identical-valued SNPs. Second, we calculate *F*-statistics on all markers and select those with the top *F* values. Finally, we perform SIR on the top markers and perform two-mean clustering on the absolute coefficients of the first SIR direction. Markers in the cluster with a higher mean are the final candidates for causal SNPs. Using the top 30 markers from the *F*-statistic, we find that the FDR is 20.8% and that the average number of answers is 4.3. We show that the proposed method is an effective and easy-to-use feature selection approach in the context of rare variants.

## Competing interests

The authors declare that there are no competing interests.

## Authors’ contributions

HW carried out the statistical analysis and drafted the manuscript. CHH provided technical support for data management. SHL and TZ participated in the design of statistical analysis and presentation of results. IH conceived of the study, designed the statistical analysis, and helped to draft the manuscript.

## References

[B1] Asimit J Zeggini E Rare variant association analysis methods for complex traits Annu Rev Genet 2010 44 293 308 10.1146/annurev-genet-102209-163421 21047260

[B2] Bansal V Libiger O Torkamani A Schork NJ Statistical analysis strategies for association studies involving rare variants Nat Rev Genet 2010 11 773 785 20940738 10.1038/nrg2867 PMC3743540

[B3] Dering C Pugh E Ziegler A Statistical analysis of rare sequence variants: an overview of collapsing methods Genet Epidemiol 2011 X suppl X X X 10.1002/gepi.20643 PMC3277891 22128052

[B4] Almasy LA Dyer TD Peralta JM Kent JW Jr Charlesworth JC Curran JE Blangero J Genetic Analysis Workshop 17 mini-exome simulation BMC Proc 2011 5 suppl 9 S2 22373155 10.1186/1753-6561-5-S9-S2 PMC3287854

[B5] Li KC Sliced inverse regression for dimension reduction (with discussion) J Am Stat Assoc 1991 86 316 342 10.2307/2290563

